# Autophagy—an underestimated coordinator of construction and destruction during plant root ontogeny

**DOI:** 10.1007/s00425-021-03668-3

**Published:** 2021-06-28

**Authors:** Natalia Wojciechowska, Kornel M. Michalak, Agnieszka Bagniewska-Zadworna

**Affiliations:** Department of General Botany, Institute of Experimental Biology, Faculty of Biology, Adam Mickiewicz University, Uniwersytetu Poznańskiego 6, 61-614 Poznań, Poland

**Keywords:** Autophagy, ATG, Root development, Phloemogenesis, Xylogenesis, Aerenchyma, Senescence, PCD

## Abstract

**Main Conclusion:**

Autophagy is a key but undervalued process in root ontogeny, ensuring both the proper development of root tissues as well as the senescence of the entire organ.

**Abstract:**

Autophagy is a process which occurs during plant adaptation to changing environmental conditions as well as during plant ontogeny. Autophagy is also engaged in plant root development, however, the limitations of belowground studies make it challenging to understand the entirety of the developmental processes. We summarize and discuss the current data pertaining to autophagy in the roots of higher plants during their formation and degradation, from the beginning of root tissue differentiation and maturation; all the way to the aging of the entire organ. During root growth, autophagy participates in the processes of central vacuole formation in cortical tissue development, as well as vascular tissue differentiation and root senescence. At present, several key issues are still not entirely understood and remain to be addressed in future studies. The major challenge lies in the portrayal of the mechanisms of autophagy on subcellular events in belowground plant organs during the programmed control of cellular degradation pathways in roots. Given the wide range of technical areas of inquiry where root-related research can be applied, including cutting-edge cell biological methods to track, sort and screen cells from different root tissues and zones of growth, the identification of several lines of evidence pertaining to autophagy during root developmental processes is the most urgent challenge. Consequently, a substantial effort must be made to ensure whether the analyzed process is autophagy-dependent or not.

**Supplementary Information:**

The online version contains supplementary material available at 10.1007/s00425-021-03668-3.

## Introduction

**Fig. 1:**
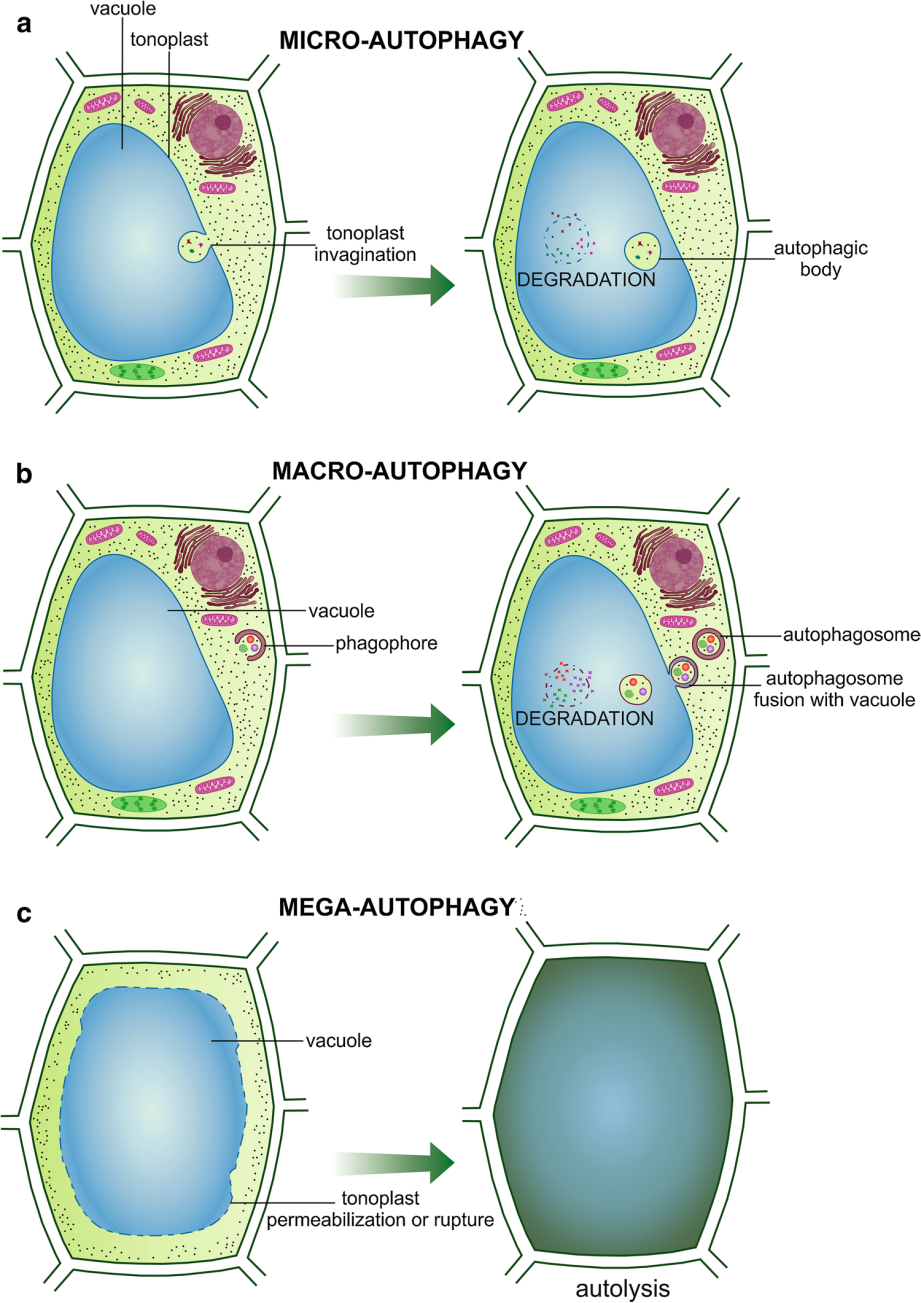
Autophagy pathways that have been confirmed in plants, micro-autophagy (**a**), where small cytoplasmic components are sequestered in the vacuole through the invagination of the tonoplast; macro-autophagy (**b**), where a double-membrane structure (phagophore) engulfs cellular material; resulting in the formation of an autophagosome that fuses with the vacuole for further degradation; mega-autophagy (**c**), where the tonoplast is permeable or ruptured; allowing the release of the lytic contents into the cytoplasm and final protoplast autolysis

Autophagy is a major pathway for the degradation of cytoplasmic material in eukaryotic cells; including macromolecules, aggregates and the degradation of entire organelles (Izumi et al. 2010; Liu et al. 2012; Wang et al. 2013; Li et al. 2014; Floyd et al. 2015; Marshall et al. 2015; Masclaux-Daubresse et al. 2020). Autophagy has been identified as the primary process for the degradation that is activated by plants subjected to environmental stress conditions (Liu et al. 2009; Pillajo et al. 2018; Shangguan et al. 2018), starvation (Sláviková et al. 2005; Goto-Yamada et al. 2019), in root hydrotropic response (Jiménez-Nopala et al. 2018), as well as during developmental events (Sláviková et al. 2005; Kwon et al. 2010; Hanamata et al. 2014; Machado and Rodrigues 2019; Wojciechowska et al. 2019), ageing and senescence (Xiong et al. 2005; Wojciechowska et al. 2018a). Even within the same plant cell, autophagy operates under favorable conditions and the autophagic activity can be upregulated under stress conditions (Slavikova et al. 2005; Fan et al. 2019). Moreover, autophagy can occur in plant cells constitutively, determining the proper development of plant organs (Inoue et al. 2006; Yano et al. 2007). Independently of the process studied, the regulation of autophagy is strictly controlled by the machinery mediated by transcription factors and epigenetic regulators (Yang et al. 2020). Autophagy may operate in a specific manner towards the degradation of specific molecules or structures, and is termed ‘selective autophagy’ (Honig et al. 2012; Yoshimoto et al. 2014; Kellner et al. 2017; Borek et al. 2019). It can also act in a general and non-selective manner; providing raw material into the vacuole (Li and Vierstra 2012; Wang et al. 2018). Besides, depending on the conditions, it can cause the removal of individual damaged or unnecessary organelles or it can be engaged in the unspecific cellular degradation. Given the above, paradoxically, as it was suggested, autophagy is involved both in cell life and cell death; playing a dual role in cell death suppression and promotion (Ustun et al. 2017) as well as cell death initiator and executor (Minina et al. 2014a). When it is involved in developmental programmed cell death (dPCD), differentiation-induced execution is tightly controlled. Molecular mechanisms governing dPCD include several steps from sequential transcriptomic reprogramming, accumulation of lytic enzymes and autophagy-related events (Phase I), to signalling and the initiation of cell death execution (Phase II) and finally completement of cell death and autolysis (Phase III) (Van Durme and Nowack 2016). Autophagic mechanisms rely on the coordinated operation for a number of cellular processes. All of these aforementioned processes were classified and previously described in detail for different processes and conditions (van Doorn and Woltering 2005; Yang et al. 2015; Goto-Yamada et al. 2019; Machado and Rodrigues 2019). Depending on whether the cell follows the terminal pathway to death or not, only some or all of the autophagic processes, such as micro-, macro- and mega-autophagy, can be initiated. Microautophagy is typically characterized by tonoplast invagination with small cytoplasmic material followed by the formation of vesicles inside the vacuole. These vesicles are termed ‘autophagic bodies’ and enable the digestion of cargo by vacuolar enzymes (Fig. 1a). Undoubtedly, however, the most common and well-characterized type of autophagy is termed macroautophagy. In plants, the macroautophagy pathway involves several steps, from induction through the regulation and generation of a phagophore. This double membrane structure functions to surround and isolate components that are targeted for degradation. The macroautophagy pathway includes the expansion of phagophores and the enclosure of targeted components for autophagosome formation and finally fusion with a vacuole (Fig. 1b). These processes occur in every cell and ensure its homeostasis. However, when the cell is introduced into the programmed death path, the decisive key type is mega-autophagy. This process involves the permeabilization or rupture of the tonoplast and the release of hydrolytic enzymes into cytoplasm for bulk digestion of the remaining protoplast (Fig. 1c). Mega-autophagy is usually associated with the final stage of programmed cell death (PCD), which occurs during tracheary element development as an example (Kwon et al. 2010; Bagniewska-Zadworna et al. 2012). Shortly thereafter, this leads to cell death characterized by an irreversible loss of cellular metabolic activity and final post-mortem autolytic cell clearance. In an attempt to comprehensively understand autophagy in a plant cell, a series of analyses at the ultrastructural, histochemical and molecular level are required; however, it must be recognized that, as it was suggested, different types of autophagy (micro-, macro- and mega-autophagy) may occur simultaneously within the same cell, or sequentially as the intensity of the dPCD progresses (van Doorn and Woltering 2005; Bagniewska-Zadworna et al. 2012, 2014b). The autophagic structures in plants, characterizing the different autophagy types, can be observed using transmission electron microscopy techniques (TEM) according to specific guidelines (van Doorn and Papini 2013; Klionsky et al. 2021; Zheng et al. 2018). These approaches with the electron microscope allow scientists to monitor both selective and non-selective autophagy at the ultrastructural level. The study of autophagy, however, typically requires “-omic” approaches; such as transcriptomics or proteomics which focus on studying specific genes termed as autophagy-related genes (*ATG*) and/or the proteins they encode (Liu et al. 2018; Jacomin et al. 2018). The core *ATG* genes were originally identified in yeast (*Saccharomyces cerevisiae*) and consist of 18 genes (Avila-Ospina et al. 2014). After that, the presence of ATG homologs was also confirmed in multiple plant species such as Arabidopsis (Thompson et al. 2005; Inoue et al. 2006), rice (Su et al. 2006; Shin et al. 2009), maize (Chung et al. 2009), barley (Sobieszczuk-Nowicka et al. 2018) or petunia (Shibuya et al. 2013). *ATG* genes encode proteins that are involved in the induction and progression autophagy process. Among these proteins, ATG8 has a crucial role which is required for the elongation and enclosure steps during autophagosome assemble (Avila-Ospina et al. 2014; Xie et al. 2008). The formation of autophagic structures can be also detected using confocal laser scanning microscopy (CLSM) and by the immunolocalization of the ATG proteins which are critical for the proper formation and function of autophagosomes as well as vesicle trafficking and vacuolar fusion with autophagosome (Pankiv et al. 2010; Ryabovol and Minibayeva 2016; Li et al. 2018). In addition to the immunolocalization based on using of specific antibodies, fluorescent fusion proteins (FFP) are becoming more increasingly used to visualize the subcellular distribution of ATG proteins (Yano et al. 2007; Phillips et al. 2008; Li et al. 2014;). The great advantage of using FFP is the in vivo localization of the studied protein, without a requirement for fixation of the biological material. This is especially important because improper fixation can generate false-negative results which are a consequence of epitope masking by the cross-linking fixation (Stadler et al. 2013). Autophagy has been studied for years in plants, however, limited work pertaining to the involvement of autophagy in developmental processes within roots has been undertaken. A three-layered system has been proposed to reveal how selective autophagy can function and influence plant development and organismal fitness: stimulus specificity, cell-type specificity and subcellular compartmentalization (Stephani and Dagdas 2020). To date, however, studies of autophagy in roots has primarily focused on deciphering the role of this process in response to abiotic stress (Liu et al. 2009; Kim et al. 2014; Zhai et al. 2016; Guan et al. 2019) and nutrient deficiency (Demidchik et al. 2018). In plants, PCD has been described and reviewed in relation to both developmental processes and in response to environmental conditions (Bagniewska-Zadworna and Arasimowicz-Jelonek 2016; Van Durme and Nowack 2016; Huysmans et al. 2017). However, given that the autophagy is not always associated with PCD, such knowledge of the occurrence of autophagy, as well as its regulation in root development-related processes, determine understanding the role of the organ which is so important for the proper physiological function of plants. In an effort to increase our knowledge in this area, this work aimed to summarize and discuss current data relating to autophagy in the roots of higher plants during their development from the stage of tissue differentiation and maturation, and all the way through to the aging of the entire organ.

## Autophagy during root ontogeny

In this report, the occurrence of autophagy during histogenesis, growth and senescent phases of root ontogenesis are discussed.

### Root tissue differentiation and maturation

In their primary stage of growth, roots show a structure that is divided into three different growth zones: a region of cell divisions with meristematic cells; a region of cell elongation with elongated cells that end their divisions; and a region of maturation, where cells differentiate into different types of specific cells of particular tissues (Evert 2006a). A cross section of primary roots reveals the three tissue systems that can be distinguished: dermal, cortex (ground tissue) and vascular tissues (Gregory 2007). During their differentiation and maturation, autophagy plays a crucial role for their establishment and further functioning. Given the wide range of technical areas of inquiry where root-related research can be applied, the identification of several lines of evidence pertaining to autophagy during root developmental processes is challenging. Consequently, substantial effort must be made to ensure whether the analyzed process is autophagy-dependent or not*.*

#### Lateral root cap

Root tips are protected and wrapped up by root cap cells that are sloughed off and continuously formed by a root cap meristem (Kumpf and Nowack 2015). The autophagy-related *AtAtg8* genes are noticeable mainly in root caps and in the region of maturation, which correspond to root areas that are associated with severe protein degradation (Slavikova et al. 2005). Different strategies of autophagy have been described in this region such as the dismantling of root caps through a release of individual metabolically active border cells that are programmed to separate from each other to the cell death and rapid autolysis (Vicre et al. 2005; Driouich et al. 2007; Plancot et al. 2013; Fendrych et al. 2014). Under favorable conditions, the autophagy-related *AtATG8* gene functions predominantly in *Arabidopsis* root caps; suggesting that the role of autophagy degradation of macromolecules in this region may enable metabolite remobilization from old to newly formed cells (Slavikova et al. 2005). Rapid PCD and cellular turnover of the lateral root cap is achieved in plants to control cap size in the growing root tips (Yadav and Helariutta 2014). Interestingly, it was suggested that synchronous bursts of cell death in lateral root cap cells release pulses of auxin to surrounding root tissues. As a result, this establishes the pattern for lateral root formation and is functionally important for primary root development and branching (Xuan et al. 2016). The last step of lateral root cap differentiation and preparation for cell death, before they fully enter the root elongation zone, is controlled at the transcriptional level by ANAC033/SOMBRERO. As a result, DNA fragmentation and tonoplast rupture, followed by cell clearance through autolytic processes involvement, were noticed (Fendrych et al. 2014). Although it is well known that cell death can be preceded by the degradation of several cytoplasmic structures, there is a lack of cytological data confirming that this process actually involves autophagy-like structures.

#### Cortical tissue differentiation

**Fig. 2:**
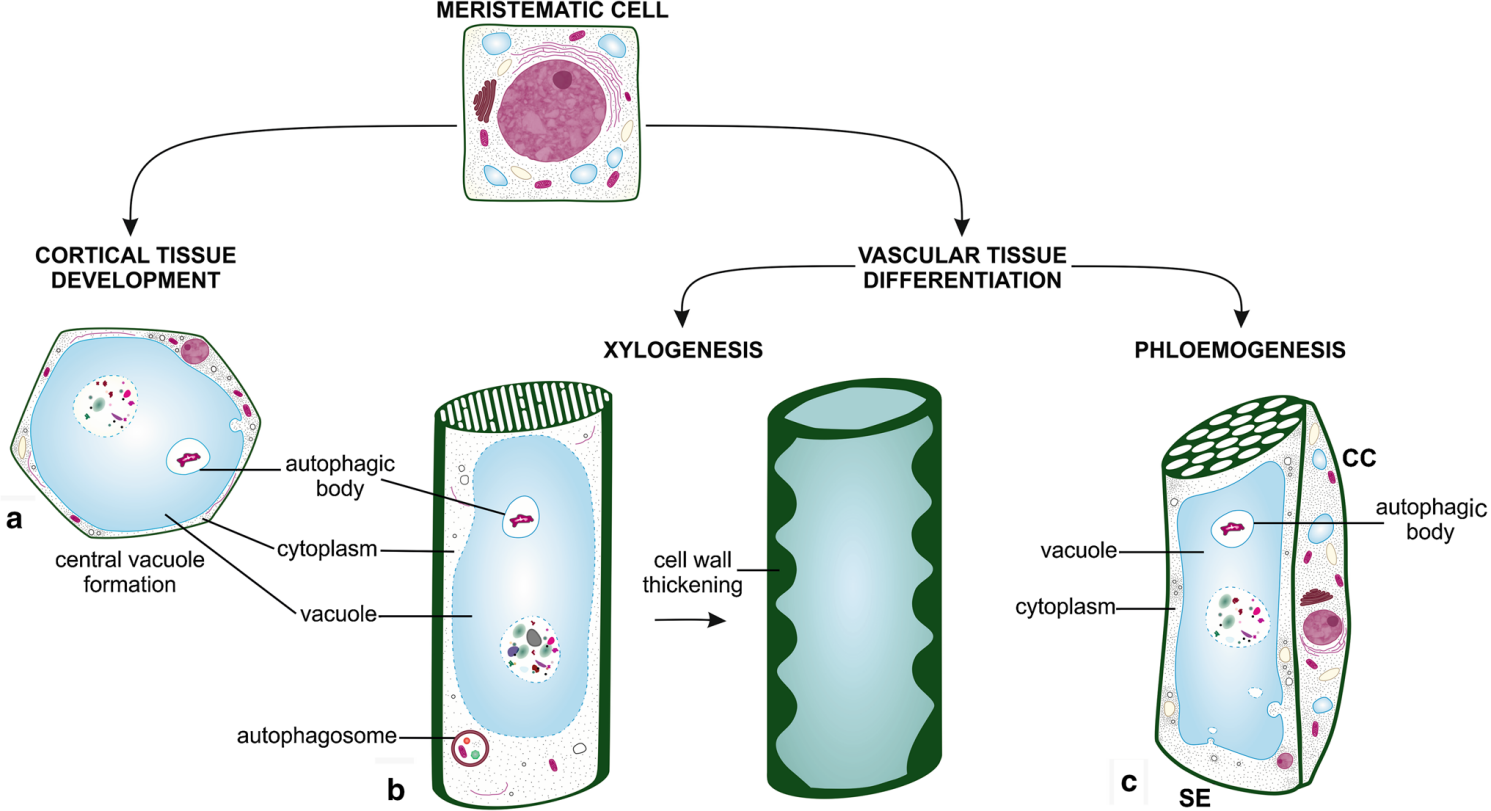
Cells, which differentiate and mature from meristematic tissue to perform specialized functions, require autophagy involvement in this process. Cortical parenchyma cells (**a**) are characterized by the presence of a large vacuole that occupies most of the cell, and the cytoplasm is only in a narrow space near the cell wall. Such structure is created through the autophagy activity and both micro- and macro-autophagy are involved in the degradation of cytoplasmic material; and this determines vacuole biogenesis to allow roots to immediately function in their role for absorption. The role of autophagy has also been proven in the differentiation of vascular tissues (**b**, **c**). During xylogenesis mature tracheary elements (TEs) are dead cells and their differentiation is related to the activation of PCD process, which involves both micro- and macro-autophagy, enabling the initial degradation of cellular components and large vacuole formation (**b**). In the last stage of xylogenesis, the tonoplast ruptures causing final autolysis. During phloemogenesis (**c**), sieve elements (SEs) undergo dramatic remodeling of its subcellular components. Mature sieve elements (SEs) lose most of their organelles while companion cells (CCs) remain highly metabolically active, with a dense cytoplasm containing the number of organelles. It is believed that autophagy might be responsible for that selective degradation of organelles in SEs. Note: the figure is not drawn to scale

There is a greater body of evidence that has been presented for the involvement of autophagy in the formation of cortical tissue. Meristematic cells, which give rise to future differentiated tissue cells, are completely filled with dense cytoplasm containing a number of organelles (Fig. 2). Cortical parenchyma cells, which have already differentiated, usually contain a large central vacuole with protoplasts located along the cell wall (Evert 2006a) (Fig. 2a). The autophagosome-like organelles in root meristematic cells were present at a very early stage of development, prior to the formation of large vacuoles (Buvat and Robert 1979; Marty 1999). In Arabidopsis and barley roots, ground tissue differentiation requires autophagy for the degradation of cytoplasmic material and proper vacuole formation from the meristematic to the elongation zone (Inoue et al. 2006). In these ground tissue cells, parts of the cytoplasm were observed to accumulate in autolytic vacuoles and pre-existing central vacuoles; suggesting that autophagy occurs constitutively in these cellular regions (Inoue et al. 2006; Yano et al. 2007). Similarly, the occurrence of constitutive autophagy was also reported as a process accompanying the formation of autolysosomes in root cells (Oh-ye et al. 2011; Merkulova et al. 2014). The number of autophagosomes in *Arabidopsis* seedling root cells increased in the elongation and maturation zones, suggesting that one of the functions of autophagy is the degradation of cytoplasmic materials for the recycling of molecules for biosynthesis (Yano et al. 2007). It was shown that constitutive autophagy occurs during the formation of the central vacuole in maturing plant cells, and is not restricted to only occurring in roots (Zouhar and Rojo 2009). Under favorable growth conditions of roots, autophagy-like structures were observed in the cytoplasm and also within the forming central vacuole (Slavikova et al. 2005). In *Arabidopsis* roots, the ATG8f protein was localized to autophagy-like structures in both the cytosol (autophagosome-resembling structures) and in the central vacuole (Slavikova et al. 2005); suggesting that the constitutive autophagy determines vacuole biogenesis also in cortical parenchyma cells. During the differentiation of cortical parenchyma cells, the precise timing of that process is critical for the elimination of the cytoplasmic material in order to allow roots to immediately function in their role for absorption.

#### Vascular tissue differentiation

Concurrent to the differentiation of cortical tissue, vascular tissue ceases to differentiate. In roots of higher plants, vascular tissues are differentiated from meristematic cells, such as procambial cells, during primary growth and vascular cambium cells during secondary growth. Two different developmental processes can be distinguished: xylogenesis and phloemogenesis, leading to xylem and phloem formation, respectively (Fig. 2b, c).

#### Xylogenesis

In cell cultures, cell death of xylem tracheary elements (TEs), which is a typical example of vacuolar cell death in dPCD, has been suggested to be autophagic (Kwon et al. 2010). The increment of vacuolization of cells undergoing dPCD was proven to be dependent on autophagy that is activated by metacaspases (Minina et al. 2013). As proposed in a hypothetical model, metacaspase type II inhibits a repressor of autophagy by cleaving it or interacting with it, which directly leads to an increased autophagic flux and a massive delivery of cytoplasmic contents into the vacuole (Minina et al. 2014b). In planta, TEs undergoing PCD involve both micro- and macro-autophagy, enabling the initial degradation of the cellular components with lytic central vacuole formation and the mega-autophagy and final autolysis after tonoplast rupture. In roots, xylogenesis proceeds from signaling via NO, through secondary cell wall synthesis and protoplast degradation that are gradual and initiated far in advance prior to lignification (Bagniewska-Zadworna et al. 2014a). These events also precede the rupture of tonoplasts and cell death. However, final autolysis, which occurs post-mortem, is the most crucial process to enable TEs to eventually function as effective conductors of nutrient fortified water, both in roots (Avci et al. 2008; Bagniewska-Zadworna et al. 2012) and in aboveground tissues (Courtois-Moreau et al. 2009; Escamez and Tuominen 2014). Interestingly, in stems, during xylem fiber differentiation, the gradual degradative processes in both the nucleus and cytoplasm occur, which results in almost a complete loss of the cytoplasmic contents well before the loss of vacuolar integrity (Courtois-Moreau et al. 2009). Thus, it seems that the development of xylem tissue must be strongly dependent on the proper functioning of autophagic as well as autolytic machineries. During the differentiation of root TEs in *Populus* (pioneer and fibrous roots), all types of autophagy have been documented to occur (Bagniewska-Zadworna et al. 2012). These processes begin with the initiation of micro- and macroautophagy along with the central vacuole formation until the formation of fully functional TEs. Autophagic-bodies within the vacuole, and the formation of autophagosomes, were demonstrated for both TEs and xylary fibers that were undergoing PCD in *Populus trichocarpa* roots (Bagniewska-Zadworna et al. 2012, 2014a; Wojciechowska et al. 2019). Autophagy-related processes appear to function both in central vacuole formation and partial degradation of cytoplasmic material at the beginning of xylem differentiation. In those differentiating cells, macroautophagy processes were initiated by a double membrane structure (phagophore) which surrounded a large portion of cytoplasm; resulting in the formation of an autophagosome after its complete enclosure of the cytoplasm. The functional mechanisms responsible for the development of autophagosomes are well understood (Kim et al. 2012); including those that function in plants (Yoshimoto and Ohsumi 2018). Proteins encoded by *ATG* genes are known to control the process of autophagosome formation by forming complexes (Yorimitsu and Klionsky 2005). Thus, the upregulation of several *ATG* genes (*ATG8C, ATG8D* and *ATG18D*) has been documented in root segments with differentiating primary and secondary xylem; while high expression of *ATG11* was exclusively noticed in roots showing secondary growth. Importantly, concurrent detection of ATG8 protein was characterized immunohistochemically during the early development of TEs and xylary fibers (Wojciechowska et al. 2019). Two forms of ATG8 protein exist, both free and conjugated to phosphatidylethanolamine (PE), which participate in the biogenesis of autophagosomes and regulation of the conjugation of ATG8 to PE and its localization to the Pre-Autophagosomal Structure (PAS) (Nair et al. 2012). Consequently, these features make the ATG8 protein a convenient molecular marker of macro-autophagy. However, it is important to note that transmission electron microscopy detected nuclei in differentiating TEs in roots until the stage at which vacuole integrity was maintained; thereby indicating that the degradation of nuclei was slow and prolonged (Bagniewska-Zadworna et al. 2014a). However, it is also proposed that TEs programmed autolysis is initiated far in advance prior to cell death but is finished post-mortem (Escamez and Tuominen 2017). It was suggested that nuclei undergo post-mortem autolysis rather than controlled degradation during the differentiation of TEs (Bollhöner et al. 2012). Similarly, autolysis is delineated as necessary for protoplast elimination in both TEs (Escamez and Tuominen 2014) and several other processes during plant development (rev by Escamez and Tuominen 2017). During TE differentiation in Arabidopsis roots, the central vacuole increases in size and xylem cysteine proteases (XCP1 and XCP2) accumulate to enable their participation in micro-autolysis within vacuoles that are still intact. After tonoplast implosion, both XCP1 and XCP2 remained associated with disintegrating cellular material of maturing TEs in roots, and degraded the bulk of the cellular contents through mega‐autolysis (Avci et al. 2008). Interestingly, barley vacuolar aspartic proteinase (phytepsin, a plant homologue to cathepsin D in animals) was detected in roots during both TE and SE development, but vacuolar cysteine proteinases were present only with TE differentiation (Runeberg‐Roos and Saarma 1998). Specifically, it is plausible that those proteinases may also play a role in active cellular degradation during xylogenesis. Due to the release of a large amount of hydrolytic enzymes from the lytic compartment, mega-autophagy followed by mega-autolysis is an irreversible step of xylem differentiation (Fig. 2b). This process occurs exactly when TEs are capable of performing their conductive function (Avci et al. 2008; Bagniewska-Zadworna et al. 2012) and after cyclosis is no longer observed after collapse of the vacuole. As a result, the last autolytic processes are also capable of occurring after the disintegration of vacuoles. Therefore, entire cell clearance can be completed after cell death (Bollhöner et al. 2013; Escamez and Tuominen 2014; Van Durme and Nowack 2016). On the other hand, as indicated by van Doorn and Woltering (2010), the rupture of the tonoplast does not necessarily mean cell death. A classic example of this scenario are phloem sieve elements which are still alive, despite the tonoplast breakage and the lack of a vacuole in their mature form. To cause death, tonoplast rupture must be followed by lytic activity of enzymes released from vacuoles. As a result, mega-autophagy is capable of causing death in most or even all examples of dPCD (van Doorn and Woltering 2010). It was shown that vacuolar cell death in the embryo suspensor of Norway spruce requires metacaspase controlled autophagy, thus cell death is not directly executed by autophagy (Minina et al. 2013). Additionally, the METACASPASE 9 (AtMC9) revealed a role in the clearance of the cell contents post-mortem, which is a crucial part of a proteolytic cascade in xylem cell death of *Arabidopsis thaliana* roots (Bollhöner et al. 2013). Interestingly, in cell cultures, TE cell types displayed higher levels of autophagy when expression of the TE-specific AtMC9 was reduced. As a consequence, this results in the modulation of autophagy to confine cell death only to the target cells (Escamez et al. 2016). It was suggested that there is a need for a tight control of autophagy in differentiating TEs undergoing PCD to implement intercellular signaling to protect surrounding cells (ectopic non-TEs) from triggering an unnecessary death path (Escamez et al. 2016). It seems plausible that there is a key to restrict cell death only to specific cell types, such as TEs.

#### Phloemogenesis

During phloemogenesis**,** it is also likely that autophagy plays a role in the partial and highly selective degradation of cytoplasmic structures from other conductive elements in roots, such as sieve elements (SEs). Unfortunately, as compared to xylogenesis, there is a lack of comprehensive literature data pertaining to the involvement and mechanisms of autophagy in the process of phloemogenesis. At their maturity, SEs do not contain many organelles and are they devoid of a nucleus, dictyosomes of the Golgi apparatus and vacuoles. The only visible intracellular components are a few plastids, mitochondria, endoplasmic reticulum, specific vesicles and phloem-specific P proteins (Eleftheriou 1996; Zhou et al. 2004; Evert 2006b; Heo et al. 2017). In contrast to dead xylary tissue, the process of cellular component degradation is slightly different in phloem. As a result, cell death does not occur and SEs remain alive and exist in a poor form with limited cellular organelles. An unusual process during the differentiation of phloem is the cessation of autophagy which ultimately leads to cell death. At the present time, the signal which triggers the sudden cessation of further degenerative processes is not known. Additionally, the molecular mechanism that is responsible for the selective degradation of cytoplasmic structures during the differentiation of phloem is not entirely understood. In recent years, several factors regulating phloem development in plants have been discovered (Truernit et al. 2012; Rodriguez-Villalon et al. 2014, 2015). However, the course and chronology of phloem differentiation has not indicated the degradation of individual structures during SE maturation. The mechanism of autophagy activity and its functional role in the process of phloemogenesis still remains to be elucidated. The majority of information for this process was obtained from the discovery of the NAC45/86 transcription factors which are responsible for the mechanism of selective autolysis of the nuclei in root SEs. Additionally, it was also discovered that this process occurred simultaneously with the autolysis of other cytoplasmic structures as well (Furuta et al. 2014). A selective autophagy-like process, similar to microautophagy, is functionally involved in the formation of phloem SEs in the developing caryopsis in wheat (Wang et al. 2008). The process pertaining to the partial degradation of cytoplasmic material in SEs is even referred to as ‘programmed cell semi-death’ (Yang et al. 2015). Within the developing caryopsis of wheat, the central vacuole and cytosol of differentiating phloem cells were found to become weakly acidified after rupture of the tonoplast (Yang et al. 2015). It is plausible that two potential degradation pathways exist; one that involves the function of the endoplasmic reticulum (ER) for the selective envelopment of organelles; and the other putative pathway involves selective inclusion into vacuoles (Wang et al. 2008). Given that the ER, and especially ER-mitochondria contact sites, is the source of membranes to drive the formation of autophagosomes (Chan and Tang 2013; Zhuang et al. 2017); it is highly probable that macroautophagy also plays a crucial role for enabling cellular degradation during phloemogenesis in root tissues as well. Accordingly, it is important to note that immunohistochemical methods confirmed the localization of the autophagy-related protein ATG8 to phloem cells exhibiting autophagy during the differentiation and early development of primary and secondary phloem; including phloem fibers in roots (Wojciechowska et al. 2019). These data provided the first premise to suggest the involvement of macroautophagy during the differentiation of phloem in roots. In contrast to the documented involvement of autophagy for xylogenesis in roots and TE differentiation where a large amount of information already exists, this has been an area of study that has been slow to progress.

**Fig. 3:**
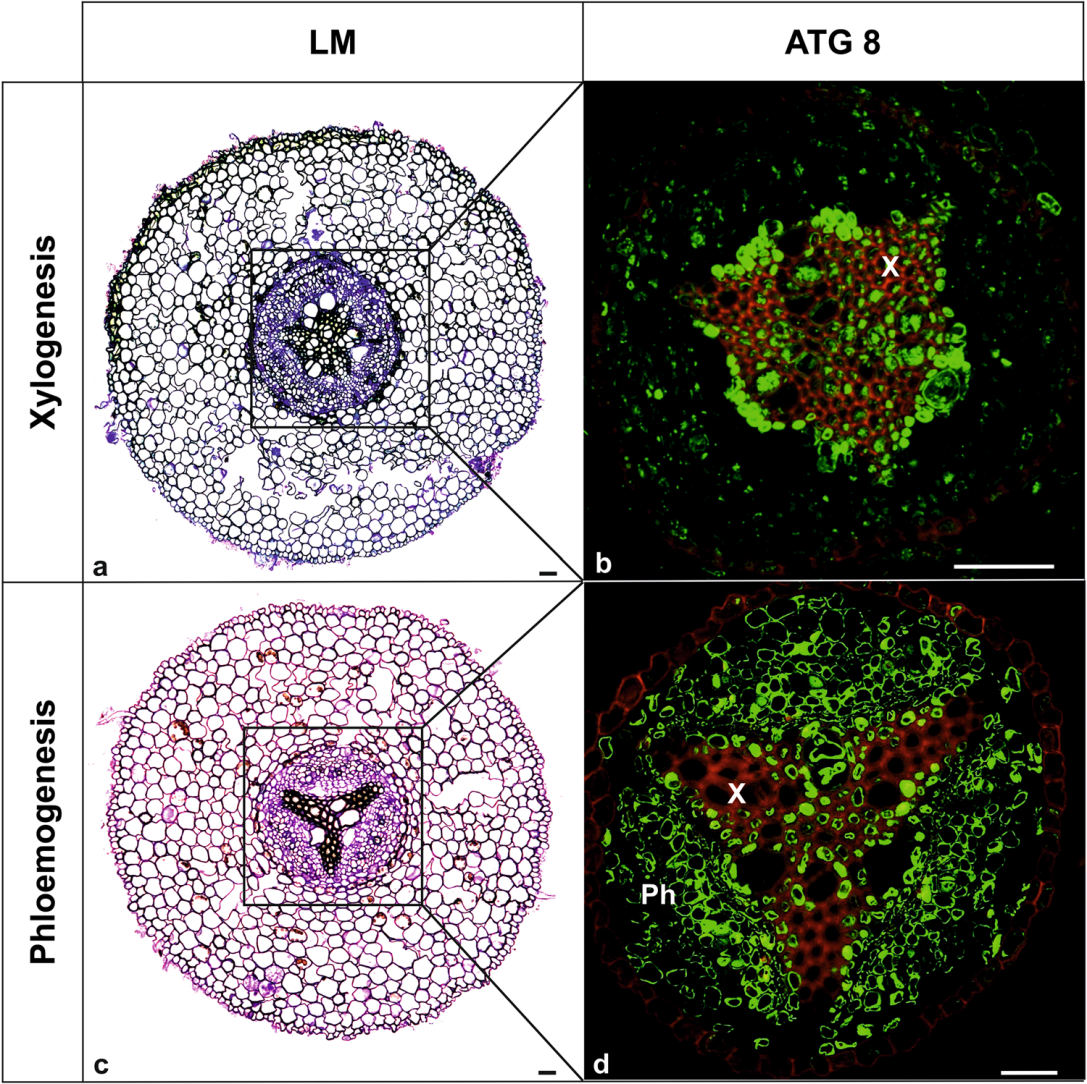
Particular tissue development was monitored at subsequent sections from the pioneer root differentiation zone of *Populus trichocarpa*. Representative images of xylem (**a**,** b**) and phloem (**c**,** d**) differentiation are presented at different distances from the root tip. In roots, the fluorescent signal appeared only in a particular root segment, exactly where particular vascular tissue differentiated. ATG8 was observed in developing primary xylem, also a strong signal came from differentiating primary phloem and from developing secondary xylem (for details see Wojciechowska et al. 2019). For each of the examples, light microscopy (LM) and ATG8 protein localization (immunofluorescence, green fluorescence, detection of both free form of ATG8 and conjugated with PE) images are provided. Lignin distribution (red autofluorescence). Methods described in Wojciechowska et al. 2019. (Original data). *Ph* phloem, *X* xylem; Bars = 50 µm

The examples detailing the involvement of autophagy in the tissue differentiation processes are presented in Fig. 3.

### Root senescence and cell death

Autophagy, which is activated as a pro-survival and pro-death process, plays a dual role in plant tissues and is actively involved in the senescence of above-ground plant organs. A significant body of work has functionally characterized the mechanisms related to autophagy and confirmed that they are active during leaf and petal senescence. Additional studies with TEM and CLSM microscopy have confirmed the processes at the ultrastructural level; as well as the analyses of *ATG* genes and ATG proteins (Guo et al. 2004; van der Graaff et al. 2006; Shibuya et al. 2011; Shibuya 2012; Shibuya et al. 2013; Avila-Ospina et al. 2014; Ishida et al. 2014; Sobieszczuk-Nowicka et al. 2018). Despite the abundance of research pertaining to the characterization of autophagy in senescing leaves and petals, only a few studies have focused on the role of autophagy in the senescence of below-ground organs. Similar to above-ground plant organs, senescence and cell death can be considered for specific root tissues (e.g., root cortical senescence; RCS), as well as for entire organs such as the seasonal senescence of fine, absorptive roots. Both processes have been classified as examples of PCD. Additionally, these processes have also been associated to similar morphological (root browning and shrinkage) and anatomical (degradation of cortex parenchyma cells) hallmarks; since known characteristics have already been documented for aboveground organs (Schneider et al. 2017; Wojciechowska et al. 2018a, b; Liu et al. 2019).

#### Root cortical senescence (RCS) and death (RCD)

**Fig. 4:**
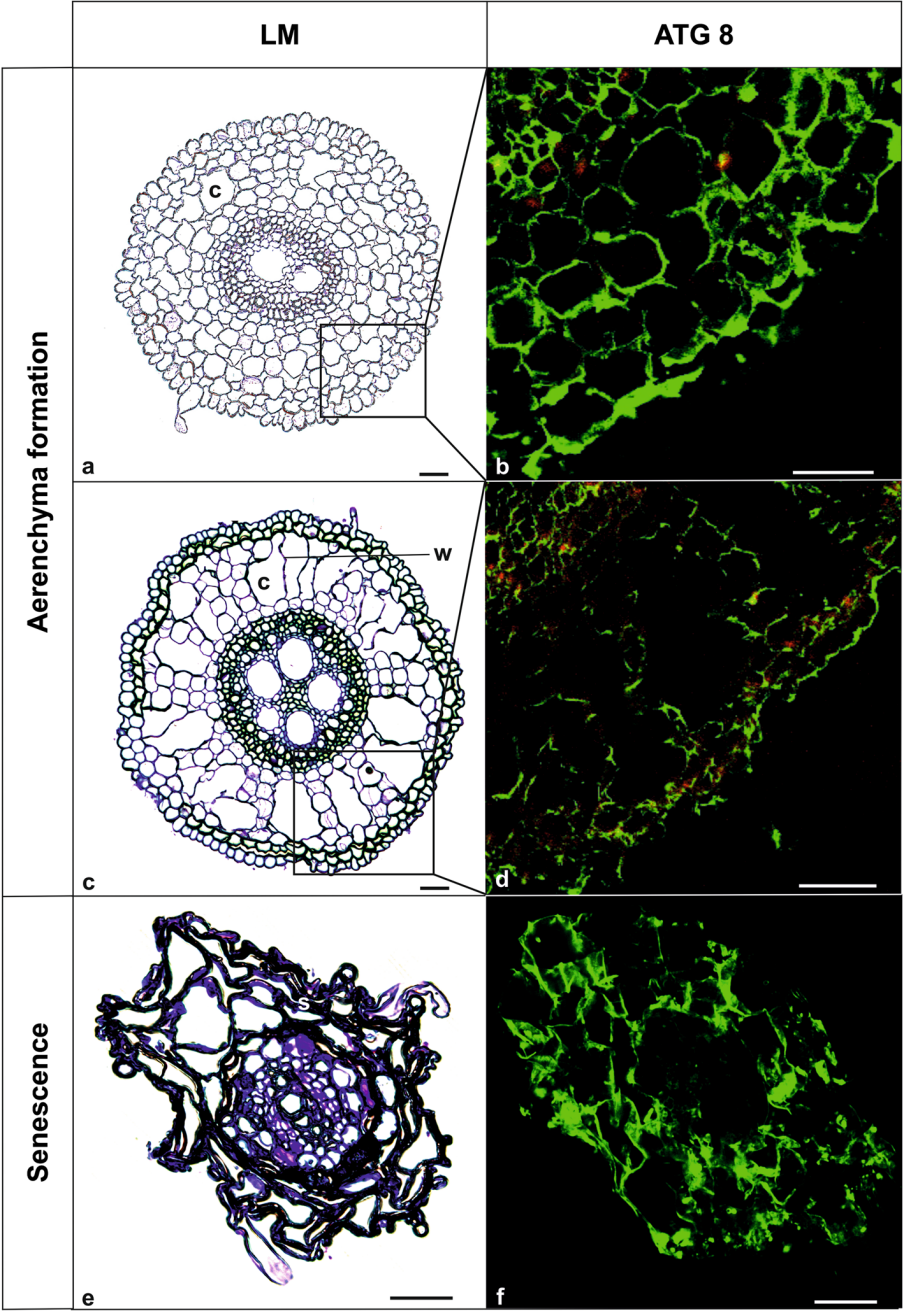
Autophagy examples in aerenchyma differentiation and root senescence. For each of the examples, light microscopy (LM) and ATG8 protein localization (immunofluorescence, green fluorescence, detection of both free form of ATG8 and conjugated with PE) images are provided. **a–d** Representative images of aerenchyma formation, which was successively monitored in the cortical cells of *Zea mays* roots grown in hydroponic conditions for 14 days. No such signal was detected in favorable conditions. Transverse section of root showing subsequent stages of lysigenous aerenchyma formation—early (**a**,** b**) and late (**c**,** d**) stages are provided. **e**,** f** Seasonal senescence of *Populus trichocarpa* fine roots. Please note the strong fluorescent signal in the cytoplasm (**b**,** d**,** f**) located along cell walls due to large central vacuole occurrence in cortical cells. Methods described in Wojciechowska et al. 2018a (Original data). *c* cortical air space, *w* wall residues of collapsed cells, *i* intact cells linking inner and outer cortex, *SC* senescent cortex. Bars (**a**–**d**) = 50 µm, (**e**–**f**) = 25 µm

RCS typically initiates in the rhizodermis and spreads towards the endodermis; especially for grass species including wheat, barley and maize (Drew et al. 2000). In accordance to what has been observed in senescent leaves, seminal roots of *Hordeum vulgare* undergo significant changes associated with cortex senescence such as the upregulation of NAC and WKRY transcription factors, increased concentration of abscisic acid (ABA) and salicylic acid (SA); and a decrease in cytokinins (CKs). Interestingly, the upregulation of *ATG* genes, which may confirm the role of autophagy in this process, has not been detected. However, the authors emphasized that seminal roots of *H. vulgare* do not contain the same abundance of proteins in comparison to leaves. Additionally, the plants were exposed to a continuous supply of nutrients which may have suppressed nutrient remobilization; and thus, the overall process of autophagy (Liu et al. 2019). It is important to note that these results are mostly relevant for annual plants; since the remobilization from roots is not as crucial as for trees. Nevertheless, it is interesting to determine the responsible mechanism for the massive degradation of cortex tissue. An interesting but not yet fully understood process pertains to the role of autophagy in the formation of aerenchyma within cortical tissue; which is referred to as root cortical death (RCD). Aerenchyma is a specialized cortical tissue which is composed of a network of interconnected gas spaces that occur in many plants to improve the aeration of the rhizosphere (Jackson and Armstrong 1999; Evans 2004). It is plausible that the formation of this tissue, which paradoxically relies on cell degradation, might be a part of normal development or a response to abiotic stress; which in most cases is directly caused by hypoxia (Evans 2004; Thomas et al. 2005). There are two basic types of aerenchyma, schizogenous and lysigenous, which differ in the pathway of origination (Evans 2004; Takahashi et al. 2014). The formation of schizogenous aerenchyma is based on cell separation and does not result in their own cell death. In contrast, however, the formation of lysigenous aerenchyma includes a series events which are similar to PCD; which ultimately lead to cell death while neighboring cells remain alive (Evans 2004). During the formation of lysigenous aerenchyma in maize roots, plasma membrane invagination, small vesicle formation, DNA fragmentation, and chromatin condensation are the examples of the PCD-related changes that have been documented (Gunawardena et al. 2001a, b). Moreover, plenty of membrane bodies enclosing organelles such as mitochondria, ER and Golgi apparatus were also observed and characterized from ultrastructural analyses. Accordingly, autophagy-like structures have also been observed during root aerenchyma formation in several species such as *Sagittaria lancifolia* (Schussler and Longstreth 2000), *Sium latifolium* (Shevchenko et al. 2016), *Triticum aestivum* (Jiang et al. 2010; Xu et al. 2013) and *Zea mays* (Gunawardena et al. 2001b; Lenochova et al. 2009). In past studies, some authors have clearly suggested that autophagy mechanisms are involved in the development of aerenchyma (Bouranis et al. 2007). However, despite their findings, there are still only a few molecular analyses which have focused on this topic. During an experiment which evaluated waterlogged roots in *Arabidopsis*, the increased expression of several *ATG* genes (*ATG2, ATG5, ATG7, ATG8e, ATG10, ATG18a*) was observed, in addition to an overall increase in the number of autophagosomes as well (Guan et al. 2019). ATG8 protein was also detected in developing aerenchyma using immunofluorescent method (Fig. 4). In addition to these aforementioned changes, a concomitant accumulation of ROS was also observed. Waterlogging is also one of the best-known stimuli of aerenchyma formation, and has also been documented to be accompanied by an increase of ROS (Xu et al. 2013; Ni et al. 2019). If we combine these data, we can hypothesize that these mechanisms function to activate autophagy in Arabidopsis roots are therefore related to the first committed step of aerenchyma formation. However, there are only a few data showing the relation of aerenchyma formation and nutrient remobilization. Some authors have suggested that root cortical aerenchyma (RCA) formation may play a dual role (1) in nutrient remobilization and (2) decreased respiration, especially in nutrient deficiencies (Adamakis et al. 2011). It was indicated that RCA may play a role in the acquisition and utilization of plant-valuable elements such as nitrogen (N), phosphorus (P), and potassium (K). It is reasonable to consider that this may be an adaptive trait to obtain nutrients by relocating them from the cortex and reducing metabolic costs of soil exploration (Adamakis et al. 2011; Postma and Lynch 2011). In nutrient-poor soils, this trait is an important factor influencing biomass and agricultural production. Moreover, it is plausible that the elevated expression of several *ATG8* genes in primary roots of maize may be related to processes that are functionally connected to the formation of aerenchyma (Li et al. 2015). To address this question, we performed immunolocalization studies which clearly documented the presence of ATG8 protein in developing aerenchyma tissue of maize roots, with stronger signal at the beginning of aerenchyma formation (Fig. 4b, d). These data provide evidence to suggest that autophagy could be involved in this process. It appears that the final stage of aerenchyma generation in roots is tonoplast rupture and cytoplasm acidification (Kawai et al. 1998; Joshi and Kumar 2012); reflecting a subsequent symptom of the implementation of mega-autophagy and autolysis.

#### The entire root senescence

At the end of the vegetative season, senescence in roots is not always connected with RCS or RCD but also with the whole organ senescence. Studies conducted on *Populus trichocarpa* have suggested that the autophagy process occurs during the senescence of fine, absorptive roots (Bagniewska-Zadworna et al. 2014b; Wojciechowska et al. 2018a). Similar to leaves and petals, these roots are classified similarly as ephemeral organs; which undergo senescence and die after performing their specific physiological functions at the whole plant level. According to previously published reports, the lifespan of these absorptive roots is species-specific and may range from a few weeks to as long as a 2-year period (Wells and Eissenstat 2001; Xia et al. 2010). Fine, absorptive roots are specifically defined as roots of the first, second and third order with a diameter smaller than 2 mm. They are also characterized by a lack of secondary structure, a high nitrogen concentration, colonization by mycorrhiza and a high surface to weight ratio (McCormack et al. 2015). Collectively, these properties make them efficient for the absorption of water and nutrients from the soil. Studies have indicated that the senescence of these roots is not a passive process and is strictly regulated; with autophagy playing a key role in the process (Wojciechowska et al. 2018a). Ultrastructural analyses have confirmed that more than one type of autophagy occurs in parenchyma cortex cells during senescence. Tonoplast invagination and the presence of autophagic bodies inside the vacuole have suggested the role of microautophagy in small cytoplasmic fragment degradation; while double-membrane vesicles with residual cytoplasmic material inside provide evidence of macroautophagy (Bagniewska-Zadworna et al. 2014b; Wojciechowska et al. 2018a). Finally, the last step of senescence, which is characterized by the rupture of tonoplasts and the degradation of cytoplasmic material after cell death, is designated as post-mortem autolytic processes. In addition to microscopic analyses, the upregulation of several *ATG8* genes (*ATG8C, ATG8D, ATG8G*) has been detected during the senescence of fine, absorptive roots (Wojciechowska et al. 2018a). *ATG8* genes encode ubiquitin-like proteins, which are required for the formation of autophagosomes and are responsible for their size regulation (Ohsumi 2001; Xie et al. 2008). In senescent roots, the amount of ATG8 protein also increased, which was similar to the elevated expression of the *ATG8* gene (Wojciechowska et al. 2018a). ATG8 protein was detected in senescent fine roots as well, especially in cortical cells, using immunofluorescent method (Fig. 4f). The changes related to the autophagy process that were documented during the senescence of absorptive, fine roots are analogous to those observed in senescent leaves. These observations indicate that autophagy is a universal process which is responsible for the proper course of senescence in ephemeral organs. Moreover, the dual role of autophagy as a pro-survival and pro-death process is emphasized during senescence (Avila-Ospina et al. 2014; Wang and Schippers 2019). During the first stage of senescence, autophagy counteracts instantaneous cell death, and maintains cell homeostasis. Additionally, autophagy also participates in the proper occurrence of the remobilization process; which is an overall key step in the senescence process (Chen et al. 2019). Gaining insight into the aging process of the roots is also essential to understand the carbon-nutrient relationships in trees (explained by Niinemets and Ostonen 2020). Consequently, the knowledge pertaining to this mechanism is crucial. Studies performed in *Populus* documented a decreased content of nitrogen along with an increased expression of genes encoding enzymes participating in N remobilization during senescence (Wojciechowska et al. 2020). However, autophagy is also one of the crucial mechanisms that is responsible for the degradation of cellular components. Functional evidence has documented that *atg* mutants are more sensitive to stress, have improper nutrient remobilization and are also characterized by premature leaf senescence and earlier cell death (Phillips et al. 2008; Guiboileau et al. 2012, 2013). Moving forward, it will be very important for the scientific community to make a comparable linkage between the senescence of fine roots in trees and the capacity of *atg* mutants to cope with premature death.

## Conclusions and perspectives

Autophagy is a crucial physiological process and its active role can be strictly related to the differentiation of tissues. As a result of this coordinated process, differentiated tissues are subsequently enabled to perform their proper biological function. In this study, the activity of autophagy during root ontogenesis was discussed; with a main focus on histogenesis, root growth and senescence. The review presents and debates multiple aspects of publicly available data. Nevertheless, these molecular processes reported in detail have not been understood in full detail at the cellular level in roots. We have shown that autophagy is potentially engaged in many root developmental processes; however, the limitations of the existing design/framework of root studies has resulted in a knowledge gap that needs to be filled. Therefore, we specifically address three topics that were not directly, or were only lightly, considered in previous works. These topics will still require further investigation to narrow down this gap: (i) the mechanism regulating the degradation of cytoplasmic structures in differentiating root cells has not yet fully elucidated, however, it is of particular interest; (ii) determining if selective autophagy may be responsible for the process of phloemogenesis in roots; (iii) characterization of a signal which may determine the abrupt cessation of degradative processes when the cell matures (e.g., cortical parenchyma cell or phloem conductive element). Scientific advancement in each of these areas will undoubtedly provide an opportunity to reconsider how roots regulate and use the autophagic machinery to ensure proper growth and functioning at the whole plant level. The research that will be necessary to address these questions can be facilitated by novel methods; a basis of which can be provided through methodological progress for immunolocalization techniques such as the use of specific antibodies, in addition to fluorescent fusion proteins (FFP) that can be implemented to visualize the subcellular distribution of autophagy-related proteins. Taken together, these approaches can ultimately provide a powerful tool and possess capacities for creating generic knowledge that can be of wider relevance; which may ultimately prove to be critical for understanding the role of autophagy in developmental processes in plants. Therefore, it is necessary to perform future investigations, using cutting-edge cell biological methods to track, sort and screen cells from different root tissues. These approaches can be used in addition to tailored strategies at the molecular level which will increase our understanding of autophagy function within particular zones of roots.

### ***Author contribution statement***

AB-Z, NW: conceptualization and original draft preparation; KM, NW: experimental data; KM: figures; AB-Z, KM, NW: review and editing.

## Supplementary Information

Below is the link to the electronic supplementary material.

Representative images of negative control reactions which omitted the primary antibody anti-ATG8. LM light microscopy image of the same pioneer root section (TIF 13048 KB)

Representative images of negative control reactions by omitting primary antibody anti-ATG8. LM light microscopy image of the same root section. **a**, **b** Aerenchyma formation in Zea mays roots, **c**, **d** Populus trichocarpa fine root senescence (TIF 7865 KB)

## References

[CR1] Adamakis IDS, Panteris E, Eleftheriou EP (2011). The fatal effect of tungsten on *Pisum**sativum* L. root cells: indications for endoplasmic reticulum stress-induced programmed cell death. Planta.

[CR2] Avci U, Petzold HE, Ismail IO, Beers EP, Haigler CH (2008). Cysteine proteases XCP1 and XCP2 aid micro-autolysis within the intact central vacuole during xylogenesis in *Arabidopsis* roots. Plant J.

[CR3] Avila-Ospina L, Moison M, Yoshimoto K, Masclaux-Daubresse C (2014). Autophagy, plant senescence, and nutrient recycling. J Exp Bot.

[CR4] Bagniewska-Zadworna A, Arasimowicz-Jelonek M (2016). The mystery of underground death: cell death in roots during ontogeny and in response to environmental factors. Plant Biol.

[CR5] Bagniewska-Zadworna A, Byczyk J, Eissenstat DM, Oleksyn J, Zadworny M (2012). Avoiding transport bottlenecks in an expanding root system: xylem vessel development in fibrous and pioneer roots under field conditions. Am J Bot.

[CR6] Bagniewska-Zadworna A, Arasimowicz-Jelonek M, Smolinski DJ, Stelmasik A (2014). New insights into pioneer root xylem development: evidence obtained from *Populus trichocarpa* plants grown under field conditions. Ann Bot.

[CR7] Bagniewska-Zadworna A, Stelmasik A, Minicka J (2014). From birth to death—*Populus trichocarpa* fibrous roots functional anatomy. Biol Plant.

[CR8] Bollhöner B, Prestele J, Tuominen H (2012). Xylem cell death: emerging understanding of regulation and function. J Exp Bot.

[CR9] Bollhöner B, Zhang B, Stael S, Denance N, Overmyer K, Goffner D, Van Breusegem F, Tuominen H (2013). Post mortem function of AtMC9 in xylem vessel elements. New Phytol.

[CR10] Borek S, Stefaniak S, Sliwinski J, Garnczarska M, Pietrowska-Borek M (2019). Autophagic machinery of plant peroxisomes. Int J Mol Sci.

[CR11] Bouranis DL, Chorianopoulou SN, Siyiannis VF, Protonotarios VE, Hawkesford MJ (2007). Lysigenous aerenchyma development in roots–triggers and cross-talks for a cell elimination program. Int J Plant Dev Biol.

[CR12] Buvat R, Robert G (1979). Vacuole formation in the actively growing root meristem of barley (*Hordeum sativum*). Am J Bot.

[CR13] Chan SN, Tang BL (2013). Location and membrane sources for autophagosome formation–from ER-mitochondria contact sites to Golgi-endosome-derived carriers. Mol Membr Biol.

[CR14] Chen Q, Shinozaki D, Luo J, Pottier M, Have M, Marmagne A, Reisdorf-Cren M, Chardon F, Thomine S, Yoshimoto K, Masclaux-Daubresse C (2019). Autophagy and nutrients management in plants. Cells.

[CR15] Chung T, Suttangkakul A, Vierstra RD (2009). The ATG autophagic conjugation system in maize: ATG transcripts and abundance of the ATG8–lipid adduct are regulated by development and nutrient availability. Plant Physiol.

[CR16] Courtois-Moreau CL, Pesquet E, Sjodin A, Muniz L, Bollhoner B, Kaneda M, Samuels L, Jansson S, Tuominen H (2009). A unique program for cell death in xylem fibers of *Populus* stem. Plant J.

[CR17] Demidchik V, Tyutereva EV, Voitsekhovskaja OV (2018). The role of ion disequilibrium in induction of root cell death and autophagy by environmental stresses. Funct Plant Biol.

[CR18] Drew MC, He CJ, Morgan PW (2000). Programmed cell death and aerenchyma formation in roots. Trends Plant Sci.

[CR19] Driouich A, Durand C, Vicre-Gibouin M (2007). Formation and separation of root border cells. Trends Plant Sci.

[CR20] Eleftheriou EP (1996). Developmental features of protophloem sieve elements in roots of wheat (*Triticum aestivum* L). Protoplasma.

[CR21] Escamez S, Tuominen H (2014). Programmes of cell death and autolysis in tracheary elements: when a suicidal cell arranges its own corpse removal. J Exp Bot.

[CR22] Escamez S, Tuominen H (2017). Contribution of cellular autolysis to tissular functions during plant development. Curr Opin Plant Biol.

[CR23] Escamez S, André D, Zhang B, Bollhöner B, Pesquet E, Tuominen H (2016). METACASPASE9 modulates autophagy to confine cell death to the target cells during Arabidopsis vascular xylem differentiation. Biol Open.

[CR24] Evans DE (2004). Aerenchyma formation. New Phytol.

[CR25] Evert RF (2006). Esau's plant anatomy, meristems, cells, and tissues of the plant body: their structure, function, and development.

[CR26] Evert RF (2006b) Phloem: cell types and developmental aspects. In: Esau's plant anatomy. Meristems, cells and tissues of the plant body: their structure, function and development. Wiley-Interscience, pp 357–406. doi: 10.1002/0470047380

[CR27] Fan J, Yu L, Xu C (2019). Dual role for autophagy in lipid metabolism in Arabidopsis. Plant Cell.

[CR28] Fendrych M, Van Hautegem T, Van Durme M, Olvera-Carrillo Y, Huysmans M, Karimi M, Lippens S, Guerin CJ, Krebs M, Schumacher K, Nowack MK (2014). Programmed cell death controlled by ANACO33/SOMBRERO determines root cap organ size in *Arabidopsis*. Curr Biol.

[CR29] Floyd BE, Morriss SC, MacIntosh GC, Bassham DC (2015). Evidence for autophagy-dependent pathways of rRNA turnover in Arabidopsis. Autophagy.

[CR30] Furuta KM, Yadav SR, Lehesranta S, Belevich I, Miyashima S, Heo JO, Vaten A, Lindgren O, De Rybel B, Van Isterdael G, Somervuo P, Lichtenberger R, Rocha R, Thitamadee S, Tahtiharju S, Auvinen P, Beeckman T, Jokitalo E, Helariutta Y (2014). Arabidopsis NAC45/86 direct sieve element morphogenesis culminating in enucleation. Science.

[CR31] Goto-Yamada S, Oikawa K, Bizan J, Shigenobu S, Yamaguchi K, Mano S, Hayashi M, Ueda H, Hara-Nishimura I, Nishimura M (2019). Sucrose starvation induces microautophagy in plant root cells. Front Plant Sci.

[CR32] Gregory P (2007). Plant roots. Wiley Online Library.

[CR33] Guan B, Lin Z, Liu D, Li C, Zhou Z, Mei F, Li J, Deng X (2019). Effect of waterlogging-induced autophagy on programmed cell death in Arabidopsis roots. Front Plant Sci.

[CR34] Guiboileau A, Yoshimoto K, Soulay F, Bataillé MP, Avice JC, Masclaux-Daubresse C (2012). Autophagy machinery controls nitrogen remobilization at the whole-plant level under both limiting and ample nitrate conditions in Arabidopsis. New Phytol.

[CR35] Guiboileau A, Avila-Ospina L, Yoshimoto K, Soulay F, Azzopardi M, Marmagne A, Lothier J, Masclaux-Daubresse C (2013). Physiological and metabolic consequences of autophagy deficiency for the management of nitrogen and protein resources in Arabidopsis leaves depending on nitrate availability. New Phytol.

[CR36] Gunawardena A, Pearce D, Jackson M, Hawes C, Evans D (2001). Rapid changes in cell wall pectic polysaccharides are closely associated with early stages of aerenchyma formation, a spatially localized form of programmed cell death in roots of maize (*Zea**mays* L.) promoted by ethylene. Plant Cell Environ.

[CR37] Gunawardena AH, Pearce DM, Jackson MB, Hawes CR, Evans DE (2001). Characterisation of programmed cell death during aerenchyma formation induced by ethylene or hypoxia in roots of maize (*Zea**mays* L.). Planta.

[CR38] Guo Y, Cai Z, Gan S (2004). Transcriptome of Arabidopsis leaf senescence. Plant Cell Environ.

[CR39] Hanamata S, Kurusu T, Kuchitsu K (2014). Roles of autophagy in male reproductive development in plants. Front Plant Sci.

[CR40] Heo JO, Blob B, Helariutta Y (2017). Differentiation of conductive cells: a matter of life and death. Curr Opi Plant Biol.

[CR41] Honig A, Avin-Wittenberg T, Galili G (2012). Selective autophagy in the aid of plant germination and response to nutrient starvation. Autophagy.

[CR42] Huysmans M, Coll NS, Nowack MK (2017). Dying two deaths—programmed cell death regulation in development and disease. Curr Opin Plant Biol.

[CR43] Inoue Y, Suzuki T, Hattori M, Yoshimoto K, Ohsumi Y, Moriyasu Y (2006). *AtATG* genes, homologs of yeast autophagy genes, are involved in constitutive autophagy in *Arabidopsis* root tip cells. Plant Cell Physiol.

[CR44] Ishida H, Izumi M, Wada S, Makino A (2014). Roles of autophagy in chloroplast recycling. Biochim Biophys Acta Biomembr.

[CR45] Izumi M, Wada S, Makino A, Ishida H (2010). The autophagic degradation of chloroplasts via rubisco-containing bodies is specifically linked to leaf carbon status but not nitrogen status in Arabidopsis. Plant Physiol.

[CR46] Jackson M, Armstrong W (1999). Formation of aerenchyma and the processes of plant ventilation in relation to soil flooding and submergence. Plant Biol.

[CR47] Jacomin A-C, Gul L, Sudhakar P, Korcsmaros T, Nezis IP (2018). What we learned from big data for autophagy research. Front Cell Dev Biol.

[CR48] Jiang Z, Song XF, Zhou ZQ, Wang LK, Li JW, Deng XY, Fan HY (2010). Aerenchyma formation: programmed cell death in adventitious roots of winter wheat (*Triticum aestivum*) under waterlogging. Funct Plant Biol.

[CR49] Jiménez-Nopala G, Salgado-Escobar AE, Cevallos-Porta D, Cárdenas L, Sepúlveda-Jiménez G, Cassab G, Porta H (2018). Autophagy mediates hydrotropic response in *Arabidopsis thaliana* roots. Plant Sci.

[CR50] Joshi R, Kumar P (2012). Lysigenous aerenchyma formation involves non-apoptotic programmed cell death in rice (*Oryza**sativa* L.) roots. Physiol Mol Biol Plants.

[CR51] Kawai M, Samarajeewa P, Barrero R, Nishiguchi M, Uchimiya H (1998). Cellular dissection of the degradation pattern of cortical cell death during aerenchyma formation of rice roots. Planta.

[CR52] Kellner R, De la Concepcion JC, Maqbool A, Kamoun S, Dagdas YF (2017). ATG8 expansion: A driver of selective autophagy diversification?. Trends Plant Sci.

[CR53] Kim S-H, Kwon C, Lee J-H, Chung T (2012). Genes for plant autophagy: functions and interactions. Mol Cells.

[CR54] Kim Y, Wang M, Bai Y, Zeng Z, Guo F, Han N, Bian H, Wang J, Pan J, Zhu M (2014). Bcl-2 suppresses activation of VPEs by inhibiting cytosolic Ca^2+^ level with elevated K^+^ efflux in NaCl-induced PCD in rice. Plant Physiol Biochem.

[CR55] Klionsky DJ, Abdel-Aziz AK, Abdelfatah S, Abdellatif M, Abdoli A, Abel S, Abeliovich H (2021). Guidelines for the use and interpretation of assays for monitoring autophagy (4th edition). Autophagy.

[CR56] Kumpf RP, Nowack MK (2015). The root cap: a short story of life and death. J Exp Bot.

[CR57] Kwon SI, Cho HJ, Jung JH, Yoshimoto K, Shirasu K, Park OK (2010). The Rab GTPase RabG3b functions in autophagy and contributes to tracheary element differentiation in *Arabidopsis*. Plant J.

[CR58] Lenochova Z, Soukup A, Votrubova O (2009). Aerenchyma formation in maize roots. Biol Plant.

[CR59] Li FQ, Vierstra RD (2012). Autophagy: a multifaceted intracellular system for bulk and selective recycling. Trends Plant Sci.

[CR60] Li F, Chung T, Vierstra RD (2014). Autophagy-related11 plays a critical role in general autophagy-and senescence-induced mitophagy in Arabidopsis. Plant Cell.

[CR61] Li F, Chung T, Pennington JG, Federico ML, Kaeppler HF, Kaeppler SM, Otegui MS, Vierstra RD (2015). Autophagic recycling plays a central role in maize nitrogen remobilization. Plant Cell.

[CR62] Li KX, Liu YN, Yu BJ, Yang WW, Yue JY, Wang HZ (2018). Monitoring autophagy in wheat living cells by visualization of fluorescence protein-tagged ATG8. Plant Cell Tissue Organ Cult.

[CR63] Liu YM, Xiong Y, Bassham DC (2009). Autophagy is required for tolerance of drought and salt stress in plants. Autophagy.

[CR64] Liu Y, Burgos JS, Deng Y, Srivastava R, Howell SH, Bassham DC (2012). Degradation of the endoplasmic reticulum by autophagy during endoplasmic reticulum stress in Arabidopsis. Plant Cell.

[CR65] Liu F, Marshall RS, Li FQ (2018). Understanding and exploiting the roles of autophagy in plants through multi-omics approaches. Plant Sci.

[CR66] Liu Z, Marella CB, Hartmann A, Hajirezaei M-R, von Wirén N (2019). An age-dependent sequence of physiological processes defines developmental root senescence. Plant Physiol.

[CR67] Machado SR, Rodrigues TM (2019). Autophagy and vacuolar biogenesis during the nectary development. Planta.

[CR68] Marshall RS, Li F, Gemperline DC, Book AJ, Vierstra RD (2015). Autophagic degradation of the 26S proteasome is mediated by the dual ATG8/ubiquitin receptor RPN10 in Arabidopsis. Mol Cell.

[CR69] Marty F (1999). Plant vacuoles. Plant Cell.

[CR70] Masclaux-Daubresse C, d'Andrea S, Bouchez I, Cacas JL (2020). Reserve lipids and plant autophagy. J Exp Bot.

[CR71] McCormack ML, Dickie IA, Eissenstat DM, Fahey TJ, Fernandez CW, Guo D, Helmisaari H-S, Hobbie EA, Iversen CM, Jackson RB, Leppälammi-Kujansuu J, Norby RJ, Phillips RP, Pregitzer KS, Pritchard SG, Rewald B, Zadworny M (2015). Redefining fine roots improves understanding of below-ground contributions to terrestrial biosphere processes. New Phytol.

[CR72] Merkulova EA, Guiboileau A, Naya L, Masclaux-Daubresse C, Yoshimoto K (2014). Assessment and optimization of autophagy monitoring methods in Arabidopsis roots indicate direct fusion of autophagosomes with vacuoles. Plant Cell Physiol.

[CR73] Minina EA, Filonova LH, Fukada K, Savenkov EI, Gogvadze V, Clapham D, Sanchez-Vera V, Suarez MF, Zhivotovsky B, Daniel G, Smertenko A, Bozhkov PV (2013). Autophagy and metacaspase determine the mode of cell death in plants. J Cell Biol.

[CR74] Minina EA, Bozhkov PV, Hofius D (2014). Autophagy as initiator or executioner of cell death. Trends Plant Sci.

[CR75] Minina EA, Smertenko AP, Bozhkov PV (2014). Vacuolar cell death in plants metacaspase releases the brakes on autophagy. Autophagy.

[CR76] Nair U, Yen W-L, Mari M, Cao Y, Xie Z, Baba M, Reggiori F, Klionsky DJ (2012). A role for Atg8–PE deconjugation in autophagosome biogenesis. Autophagy.

[CR77] Ni X-L, Gui M-Y, Tan L-L, Zhu Q, Liu W-Z, Li C-X (2019). Programmed cell death and aerenchyma formation in water-logged sunflower stems and its promotion by ethylene and ROS. Front Plant Sci.

[CR78] Niinemets Ü, Ostonen I (2020). Plant organ senescence above- and belowground in trees: how to best salvage resources for new growth?. Tree Physiol.

[CR79] Ohsumi Y (2001). Ubiquitin and proteasomes: Molecular dissection of autophagy: two ubiquitin-like systems. Nat Rev Mol.

[CR80] Oh-ye Y, Inoue Y, Moriyasu Y (2011). Detecting autophagy in Arabidopsis roots by membrane-permeable cysteine protease inhibitor E-64d and endocytosis tracer FM4-64. Plant Signal Behav.

[CR81] Pankiv S, Alemu EA, Brech A, Bruun J-A, Lamark T, Øvervatn A, Bjørkøy G, Johansen T (2010). FYCO1 is a Rab7 effector that binds to LC3 and PI3P to mediate microtubule plus end–directed vesicle transport. J Cell Biol.

[CR82] Phillips AR, Suttangkakul A, Vierstra RD (2008). The ATG12-conjugating enzyme ATG10 is essential for autophagic vesicle formation in *Arabidopsis thaliana*. Genetics.

[CR83] Pillajo JOQ, Chapin LJ, Jones ML (2018). Senescence and abiotic stress induce expression of autophagy-related genes in *Petunia*. J Am Soc Hortic Sci.

[CR84] Plancot B, Santaella C, Jaber R, Kiefer-Meyer MC, Follet-Gueye M-L, Leprince J, Gattin I, Souc C, Driouich A, Vicre-Gibouin M (2013). Deciphering the responses of root border-like cells of *Arabidopsis* and flax to pathogen-derived elicitors. Plant Physiol.

[CR85] Postma JA, Lynch JP (2011). Theoretical evidence for the functional benefit of root cortical aerenchyma in soils with low phosphorus availability. Annf Bot.

[CR86] Rodriguez-Villalon A, Gujas B, Kang YH, Breda AS, Cattaneo P, Depuydt S, Hardtke CS (2014). Molecular genetic framework for protophloem formation. PNAS.

[CR87] Rodriguez-Villalon A, Gujas B, van Wijk R, Munnik T, Hardtke CS (2015). Primary root protophloem differentiation requires balanced phosphatidylinositol-4,5-biphosphate levels and systemically affects root branching. Development.

[CR88] Runeberg-Roos P, Saarma M (1998). Phytepsin, a barley vacuolar aspartic proteinase, is highly expressed during autolysis of developing tracheary elements and sieve cells. Plant J.

[CR89] Ryabovol VV, Minibayeva FV (2016). Molecular mechanisms of autophagy in plants: Role of ATG8 proteins in formation and functioning of autophagosomes. Biochemistry.

[CR90] Schneider HM, Postma JA, Wojciechowski T, Kuppe C, Lynch JP (2017). Root cortical senescence improves growth under suboptimal availability of N, P, and K. Plant Physiol.

[CR91] Schussler EE, Longstreth DJ (2000). Changes in cell structure during the formation of root aerenchyma in S*agittaria lancifolia* (*Alismataceae*). Am J Bot.

[CR92] Shangguan LF, Fang X, Chen LD, Cui LW, Fang JG (2018). Genome-wide analysis of autophagy-related genes (ARGs) in grapevine and plant tolerance to copper stress. Planta.

[CR93] Shevchenko G, Brykov V, Ivanenko G (2016). Specific features of root aerenchyma formation in *Sium**latifoliun* L. (*Apiaceae*). Cytol Genet.

[CR94] Shibuya K (2012). Molecular mechanisms of petal senescence in ornamental plants. J Jpn Soc Hortic Sci.

[CR95] Shibuya K, Shimizu K, Yamada T, Ichimura K (2011). Expression of autophagy-associated ATG8 genes during petal senescence in Japanese morning glory. J Jpn Soc Hortic Sci.

[CR96] Shibuya K, Niki T, Ichimura K (2013). Pollination induces autophagy in petunia petals via ethylene. J Exp Bot.

[CR97] Shin J-H, Yoshimoto K, Ohsumi Y, Jeon J-s, An G (2009). OsATG10b, an autophagosome component, is needed for cell survival against oxidative stresses in rice. Mol Cells.

[CR98] Sláviková S, Shy G, Yao Y, Glozman R, Levanony H, Pietrokovski S, Elazar Z, Galili G (2005). The autophagy-associated ATG8 gene family operates both under favourable growth conditions and under starvation stresses in Arabidopsis plants. J Exp Bot.

[CR99] Sobieszczuk-Nowicka E, Wrzesiński T, Bagniewska-Zadworna A, Kubala S, Rucińska-Sobkowiak R, Polcyn W, Misztal L, Mattoo AK (2018). Physio-genetic dissection of dark-induced leaf senescence and timing its reversal in barley. Plant Physiol.

[CR100] Stadler C, Rexhepaj E, Singan VR, Murphy RF, Pepperkok R, Uhlén M, Simpson JC, Lundberg E (2013). Immunofluorescence and fluorescent-protein tagging show high correlation for protein localization in mammalian cells. Nat Methods.

[CR101] Stephani M, Dagdas Y (2020). Plant selective autophagy-still an uncharted territory with a lot of hidden gems. J Mol Biol.

[CR102] Su W, Ma H, Liu C, Wu J, Yang J (2006). Identification and characterization of two rice autophagy associated genes, OsAtg8 and OsAtg4. Mol Biol Rep.

[CR103] Takahashi H, Yamauchi T, Colmer TD, Nakazono M (2014) Aerenchyma formation in plants. In: Low-Oxygen Stress in Plants. Springer, pp 247–265. doi:10.1007/978-3-7091-1254-0_13

[CR104] Thomas A, Guerreiro S, Sodek L (2005). Aerenchyma formation and recovery from hypoxia of the flooded root system of nodulated soybean. Ann Bot.

[CR105] Thompson AR, Doelling JH, Suttangkakul A, Vierstra RD (2005). Autophagic nutrient recycling in Arabidopsis directed by the ATG8 and ATG12 conjugation pathways. Plant Physiol.

[CR106] Truernit E, Baub H, Belcram K, Barthelemy J, Palauqui JC (2012). OCTOPUS, a polarly localised membrane-associated protein, regulates phloem differentiation entry in *Arabidopsis thaliana*. Development.

[CR107] Ustun S, Hafren A, Hofius D (2017). Autophagy as a mediator of life and death in plants. Curr Opin Plant Biol.

[CR108] van der Graaff E, Schwacke R, Schneider A, Desimone M, Flügge U-I, Kunze R (2006). Transcription analysis of Arabidopsis membrane transporters and hormone pathways during developmental and induced leaf senescence. Plant Physiol.

[CR109] van Doorn WG, Papini A (2013). Ultrastructure of autophagy in plant cells: a review. Autophagy.

[CR110] van Doorn WG, Woltering EJ (2005). Many ways to exit? Cell death categories in plants. Trends Plant Sci.

[CR111] van Doorn WG, Woltering EJ (2010). What about the role of autophagy in PCD?. Trends Plant Sci.

[CR112] Van Durme M, Nowack MK (2016). Mechanisms of developmentally controlled cell death in plants. Curr Opin Plant Biol.

[CR113] Vicre M, Santaella C, Blanchet S, Gateau A, Driouich A (2005). Root border-like cells of *Arabidopsis*. Microscopical characterization and role in the interaction with rhizobacteria. Plant Physiol.

[CR114] Wang H, Schippers JH (2019). The role and regulation of autophagy and the proteasome during aging and senescence in plants. Genes.

[CR115] Wang L, Zhou Z, Song X, Li J, Deng X, Mei F (2008). Evidence of ceased programmed cell death in metaphloem sieve elements in the developing caryopsis of *Triticum aestivum* L. Protoplasma.

[CR116] Wang Y, Yu B, Zhao J, Guo J, Li Y, Han S, Huang L, Du Y, Hong Y, Tang D (2013). Autophagy contributes to leaf starch degradation. Plant Cell.

[CR117] Wang P, Mugume Y, Basshama DC (2018). New advances in autophagy in plants: regulation, selectivity and function. Semin Cell Dev Biol.

[CR118] Wells CE, Eissenstat DM (2001). Marked differences in survivorship among apple roots of different diameters. Ecology.

[CR119] Wojciechowska N, Marzec-Schmidt K, Kalemba EM, Zarzyńska-Nowak A, Jagodziński AM, Bagniewska-Zadworna A (2018). Autophagy counteracts instantaneous cell death during seasonal senescence of the fine roots and leaves in *Populus trichocarpa*. BMC Plant Biol.

[CR120] Wojciechowska N, Sobieszczuk-Nowicka E, Bagniewska-Zadworna A (2018). Plant organ senescence - regulation by manifold pathways. Plant Biol.

[CR121] Wojciechowska N, Smugarzewska I, Marzec-Schmidt K, Zarzyńska-Nowak A, Bagniewska-Zadworna A (2019). Occurrence of autophagy during pioneer root and stem development in *Populus trichocarpa*. Planta.

[CR122] Wojciechowska N, Marzec-Schmidt K, Kalemba EM, Ludwików A, Bagniewska-Zadworna A (2020). Seasonal senescence of leaves and roots of *Populus trichocarpa*—is the scenario the same or different?. Tree Physiol.

[CR123] Xia M, Guo D, Pregitzer KS (2010). Ephemeral root modules in *Fraxinus mandshurica*. New Phytol.

[CR124] Xie Z, Nair U, Klionsky DJ (2008). Atg8 controls phagophore expansion during autophagosome formation. Mol Biol Cell.

[CR125] Xiong Y, Contento AL, Bassham DC (2005). AtATG18a is required for the formation of autophagosomes during nutrient stress and senescence in *Arabidopsis thaliana*. Plant J.

[CR126] Xu QT, Yang L, Zhou ZQ, Mei FZ, Qu LH, Zhou GS (2013). Process of aerenchyma formation and reactive oxygen species induced by waterlogging in wheat seminal roots. Planta.

[CR127] Xuan W, Band LR, Kumpf RP, Van Damme D, Parizot B, De Rop G, Opdenacker D, Möller BK, Skorzinski N, Njo MF (2016). Cyclic programmed cell death stimulates hormone signaling and root development in Arabidopsis. Science.

[CR128] Yadav SR, Helariutta Y (2014). Programmed cell death: new role in trimming the root tips. Curr Biol.

[CR129] Yang WL, Cai JT, Zhou ZQ, Zhou GS, Mei FZ, Wang LK (2015). Microautophagy involves programmed cell semi-death of sieve elements in developing caryopsis of *Triticum aestivum* L. Cell Biol Int.

[CR130] Yang C, Luo M, Zhuang XH, Li FQ, Gao CJ (2020). Transcriptional and epigenetic regulation of autophagy in plants. Trends Genet.

[CR131] Yano K, Suzuki T, Moriyasu Y (2007). Constitutive autophagy in plant root cells. Autophagy.

[CR132] Yorimitsu T, Klionsky DJ (2005). Autophagy: molecular machinery for self-eating. Cell Death Differ.

[CR133] Yoshimoto K, Ohsumi Y (2018). Unveiling the molecular mechanisms of plant autophagy-from autophagosomes to vacuoles in plants. Plant Cell Physiol.

[CR134] Yoshimoto K, Shibata M, Kondo M, Oikawa K, Sato M, Toyooka K, Shirasu K, Nishimura M, Ohsumi Y (2014). Organ-specific quality control of plant peroxisomes is mediated by autophagy. J Cell Sci.

[CR135] Zhai YF, Guo M, Wang H, Lu JP, Liu JH, Zhang C, Gong ZH, Lu MH (2016). Autophagy, a conserved mechanism for protein degradation, responds to heat, and other abiotic stresses in *Capsicum annuum* L. Front Plant Sci.

[CR136] Zheng X, Zhao C, Liu Y (2018). Examining autophagy in plant by Transmission Electron Microscopy (TEM). Bio-Protoc.

[CR137] Zhou Z-Q, Lan S-Y, Zhu X-T, Wang W-J, Xu Z-X (2004). Ultrastructure and its function of phloem cell in abdominal vascular bundle of wheat caryopsis. Acta Agron Sin.

[CR138] Zhuang X, Chung KP, Cui Y, Lin W, Gao C, Kang B-H, Jiang L (2017). ATG9 regulates autophagosome progression from the endoplasmic reticulum in Arabidopsis. PNAS.

[CR139] Zouhar J, Rojo E (2009). Plant vacuoles: where did they come from and where are they heading?. Curr Opin Plant Biol.

