# Supplementary figures and images for: Autophagy—an underestimated coordinator of construction and destruction during plant root ontogeny
Source: Planta. 2021 Jun 28;254(1):15. doi: 10.1007/s00425-021-03668-3 (PMC8238727; doi:10.1007/s00425-021-03668-3)

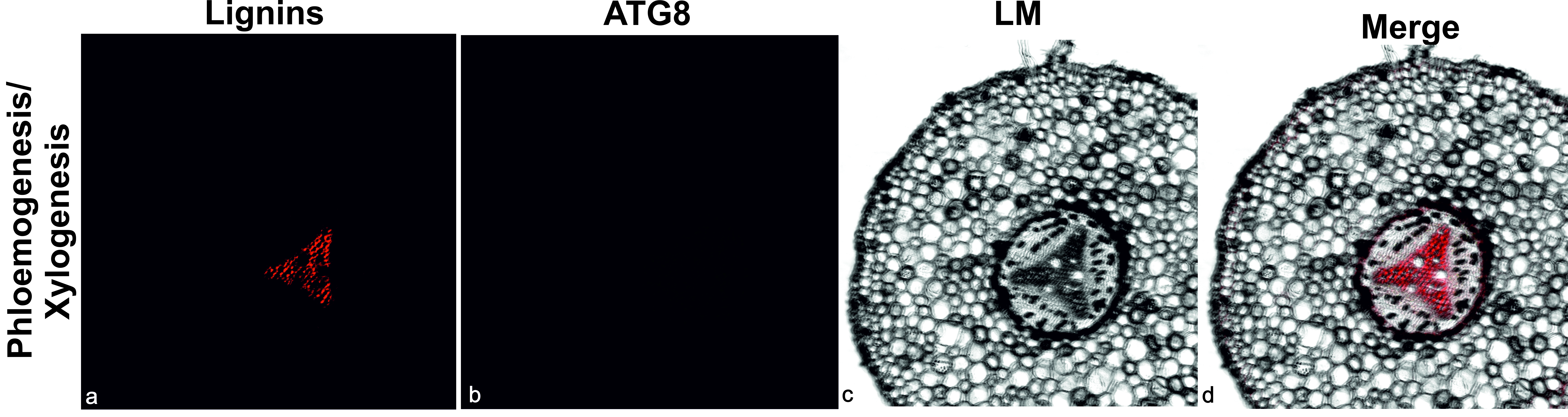

Supplement: Supplementary file 1 — Representative images of negative control reactions which omitted the primary antibody anti-ATG8. LM light microscopy image of the same pioneer root section (TIF 13048 KB) [file 425_2021_3668_MOESM1_ESM.tif]

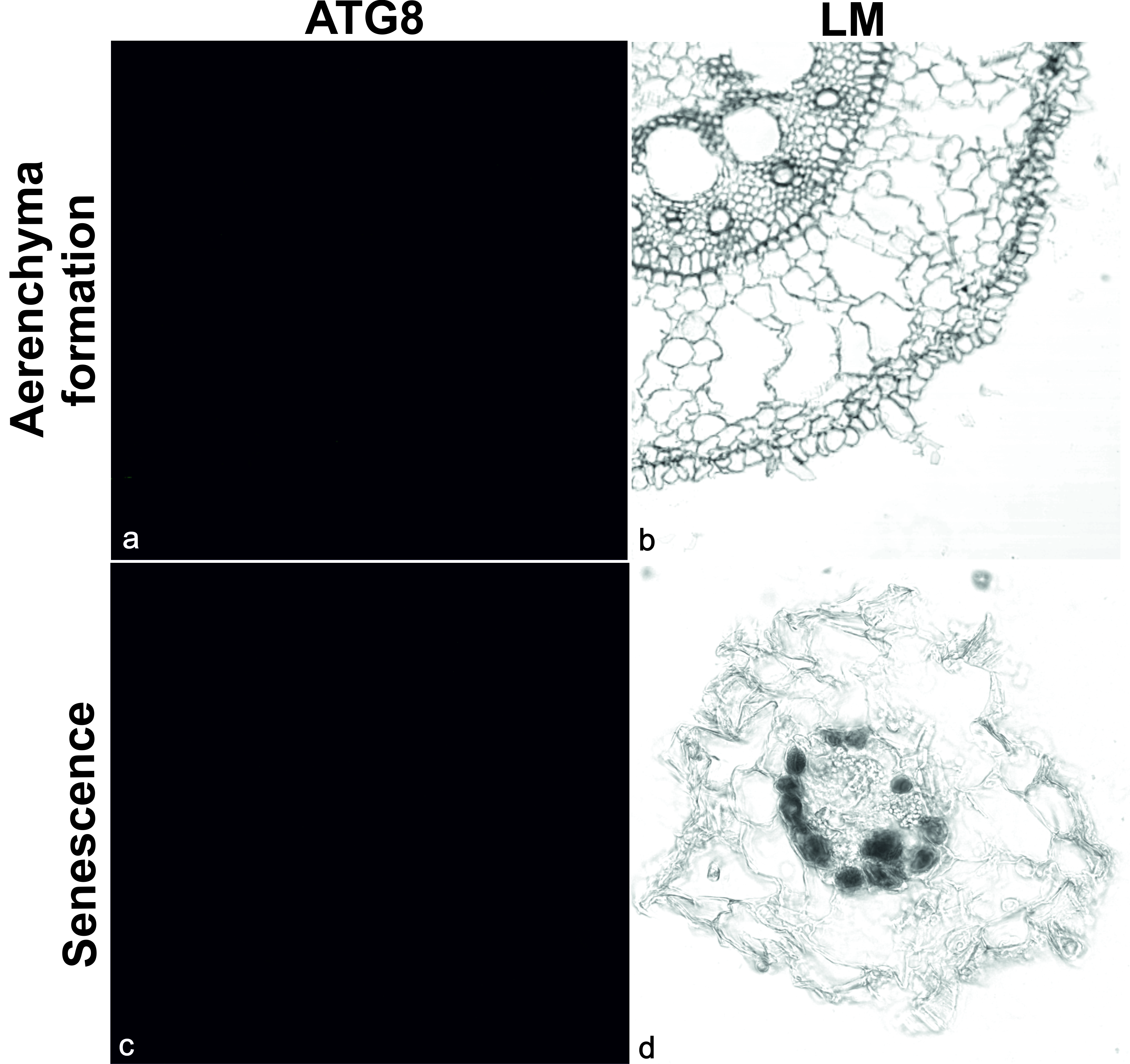

Supplement: Supplementary file 2 — Representative images of negative control reactions by omitting primary antibody anti-ATG8. LM light microscopy image of the same root section. a, b Aerenchyma formation in Zea mays roots, c, d Populus trichocarpa fine root senescence (TIF 7865 KB) [file 425_2021_3668_MOESM2_ESM.tif]
